# Phase Diagram and High-Temperature Superconductivity of Compressed Selenium Hydrides

**DOI:** 10.1038/srep15433

**Published:** 2015-10-22

**Authors:** Shoutao Zhang, Yanchao Wang, Jurong Zhang, Hanyu Liu, Xin Zhong, Hai-Feng Song, Guochun Yang, Lijun Zhang, Yanming Ma

**Affiliations:** 1 State Key Laboratory of Superhard Materials, Jilin University, Changchun 130012, China; 2 Faculty of Chemistry, Northeast Normal University, Changchun 130024, China.; 3 College of Materials Science and Engineering and Key Laboratory of Automobile Materials of MOE, Jilin University, Changchun 130012, China; 4 LCP, Institute of Applied Physics and Computational Mathematics, Beijing 100088, China; 5 Software Center for High Performance Numerical Simulation, China Academy of Engineering Physics, Beijing 100088, China

## Abstract

Recent discovery of high-temperature superconductivity (*T*_*c*_ = 190 K) in sulfur hydrides at megabar pressures breaks the traditional belief on the *T*_*c*_ limit of 40 K for conventional superconductors, and opens up the doors in searching new high-temperature superconductors in compounds made up of light elements. Selenium is a sister and isoelectronic element of sulfur, with a larger atomic core and a weaker electronegativity. Whether selenium hydrides share similar high-temperature superconductivity remains elusive, but it is a subject of considerable interest. First-principles swarm structure predictions are performed in an effort to seek for energetically stable and metallic selenium hydrides at high pressures. We find the phase diagram of selenium hydrides is rather different from its sulfur analogy, which is indicated by the emergence of new phases and the change of relative stabilities. Three stable and metallic species with stoichiometries of HSe_2_, HSe and H_3_Se are identified above ~120 GPa and they all exhibit superconductive behaviors, of which the hydrogen-rich HSe and H_3_Se phases show high *T*_*c*_ in the range of 40–110 K. Our simulations established the high-temperature superconductive nature of selenium hydrides and provided useful route for experimental verification.

Since Onnes discovered superconductivity of mercury in 1911^1^, intensive research activities were stimulated to search for new superconductors with high critical temperatures (*T*_c_). With this thrust, unconventional superconductors such as cuprates^2^,^3^,^4^ and Fe-pnictides^5^,^6^,^7^, in which superconducting mechanism cannot be described by Bardeen-Cooper-Schrieffer (BCS) theory^8^ have attracted major attention since they often exhibit high *T*_c_ values, reaching as high as 164 K (at high pressures)^9^. In spite of extensive research for several decades, the superconducting mechanism in these unconventional superconductors is still controversial, preventing them from being an optimal platform for designing the superconductors with the higher *T*_c_.

On the side of conventional superconductors, the situation is rather disappointing since they all have low *T*_c_ values. The best-known conventional superconductor of MgB_2_ has the *T*_c_ of 39 K^10^. As such, there is a traditional belief on the *T*_c_ limit of 40 K for conventional superconductors in the field.

BCS theory gives a clear count on superconducting mechanism of conventional superconductors, making the design of high-*T*_*c*_ superconductors possible. According to BCS theory, a necessary condition for a high-*T*_*c*_ superconductor is that the metallic compounds shall have large electron density of states at the Fermi level, high phonon frequencies and strong electron-phonon coupling. Hydrogen is the lightest element, and therefore naturally gives rise to high phonon frequencies, and also owes the unique strong bare electron-ion interaction. Ashcroft firstly proposed that solid hydrogen once being metallic under pressure has the potential to be a high-temperature superconductor^11^. Later on, the idea on metallic superconducting hydrogen was extended into hydrogen-rich compounds^12^, where metallization pressure can be significantly lowered than that in pure hydrogen. A number of hydrogen-rich compounds were subsequently predicted to be good superconductors^13^ with estimated *T*_c_ reaching remarkably high values (*e.g.* 64 K for GeH_4_ at 220 GPa^14^, 107 K for SiH_4_(H_2_)_2_ at 250 GPa^15^, 235 K for CaH_6_ at 150 GPa^16^, and 204 K for (H_2_S)_2_H_2_ at 200 GPa^17^). Experimental syntheses of these potential high-temperature superconductors are excitingly ongoing, but challenging.

Sulfur dihydride (H_2_S) has not been considered as the candidate for superconducting hydrides since it was proposed to dissociation into elemental sulfur and hydrogen before the metallization^18^,^19^. Only recently, first-principles swarm structure searches on high-pressure structures of H_2_S was conducted and H_2_S was excluded from the elemental dissociation, making the prediction of its superconductivity with a high *T*_c_ ~80 K at 160 GPa possible^20^. Shortly after this report, breakthrough electrical measurement observed high-temperature superconductivity in compressed H_2_S with an unprecedentedly high *T*_c_ up to 190 K at megabar pressures^21^. The decrease of *T*_c_ with magnetic field, and the strong isotope shift of *T*_c_ suggest that H_2_S is a conventional superconductor. There exist two observed superconducting states: the sample prepared at low temperature of 100–150 K has a maximal *T*_c_ of 150 K at 200 GPa, while the 190 K superconductivity comes from the sample prepared at high temperature of 220–300 K, which likely associates with the dissociation of H_2_S into H_3_S^21^,^22^,^23^. This discovery has stimulated significant interest in studying the underlying superconducting mechanism^23^,^24^,^25^,^26^,^27^ and searching for new high-*T*_c_ superconductors in other dense hydride systems.

Selenium is a sister and isoelectronic element of sulfur with a larger atomic radius and a weaker electronegativity. By witness of high-*T*_c_ superconductivity of sulfur hydrides (*i.e.* H-S system), a natural and immediate thought is to examine whether selenium hydrides (H-Se system) are also high-temperature superconductors at high pressures. To our best knowledge, there is less report on solid phases of H-Se system, except for the existence of three temperature-dependent H_2_Se phases at ambient pressure^28^.

We herein explored the hitherto unknown high-pressure phase diagram of the H-Se system via the first-principles swarm-intelligence based structure search. In the moderate pressure region, different from the H-S system all the H-Se compounds are energetically unstable against the elemental decomposition including the normally expectant H_2_Se stoichiometry. At megabar pressures above 120 GPa, three stable species with stoichiometries of HSe_2_, HSe and H_3_Se were identified. Among them HSe with the high-symmetry *P*4/*nmm* structure is the most stable phase and H_3_Se becomes marginally stable at high pressures. They are all metallic and exhibit superconductive behaviors. The latter two H-rich phases, benefiting from fairly strong electron-phonon coupling, are predicted to owe high *T*_*c*_ values up to 110 K. Our simulations provide a useful roadmap for discovering high-temperature superconductors in selenium hydrides.

## Results

**Figure 1:**
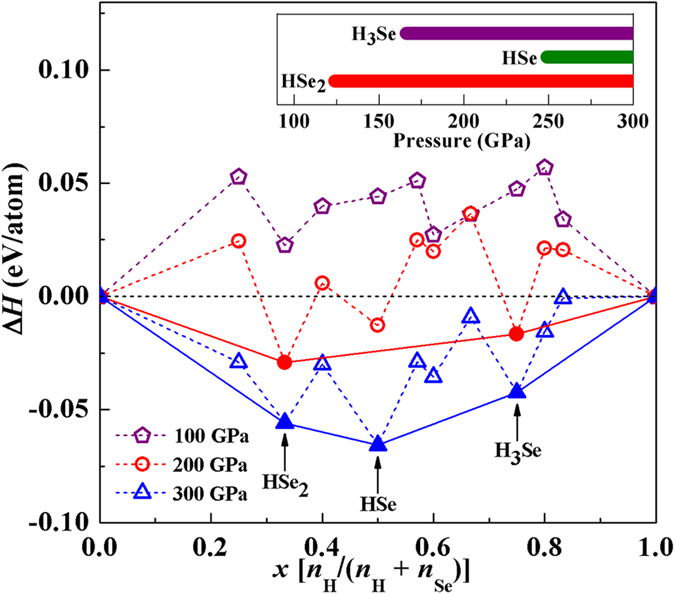
Calculated formation enthalpies (*H* in eV/atom) of various selenium hydrides with respect to the elemental decomposition into solidified H_2_ and Se at 100 (violet), 200 (red) and 300 (blue) GPa, respectively. At each stoichiometry, only *H* of the lowest-energy structure is shown. The phase IV (*C*2*/m*) and phase VI (*Im-*3*m*) of Se^30^, the *P*6_3_*m* and *C*2/*c* structure of solid H_2_ in respective stable pressure regions are chosen for calculating *H*. The inset plot shows the pressure range in which each stable stoichiometry is stabilized.

The variable-composition structure searches are performed at a variety of H-Se stoichiometries containing up to 4 formula units per simulation cell at 0, 50, 100, 200 and 300 GPa. At 0 GPa, we find only one stable stoichiometry with respect to the elemental decomposition, H_2_Se (see Supplemental Fig. S1a), which is consistent with available experimental reports^28^. It is in the *P*3_1_21 symmetry, where the arrangement of Se atoms is nearly the same as that of the Se-I phase^29^ and H atoms are accommodated on the line of two adjacent Se to form covalent H-Se bonds and hydrogen bonds simultaneously (Fig. S1c). However, with increasing pressure this phase becomes dramatically unstable (Fig. S1b). The structure search results are summarized in the convex hulls constructed with solid H_2_ and Se as the binary variables in Fig. 1 (and Fig. S1a). In the low-pressure region up to 100 GPa, all the stoichiometries are energetically unstable against the elemental decomposition. This is in sharp contrast to the H-S system, where all the investigated stoichiometries are stable with respect to the elemental decomposition^22^. With increasing pressure to 200 GPa, while most of phases still lie above the elemental decomposition line, three stoichiometries (HSe_2_, HSe and H_3_Se) show the tendency of being stabilized and move downward below the line. Eventually HSe_2_ and H_3_Se become stable stoichiometries on the hull against any way of decomposition. At 300 GPa, the HSe_2_, HSe and H_3_Se stoichiometries are all clearly located on the hull, and HSe emerges as the most stable phase over other species. The energetic instability of these phases in the low-pressure region and their being restabilized at high pressures may be attributed to the weaker covalent bond of H-Se than that of H-S (resulted from the larger size of Se), which is cumulatively strengthened by volume contraction under compression.

**Figure 2:**
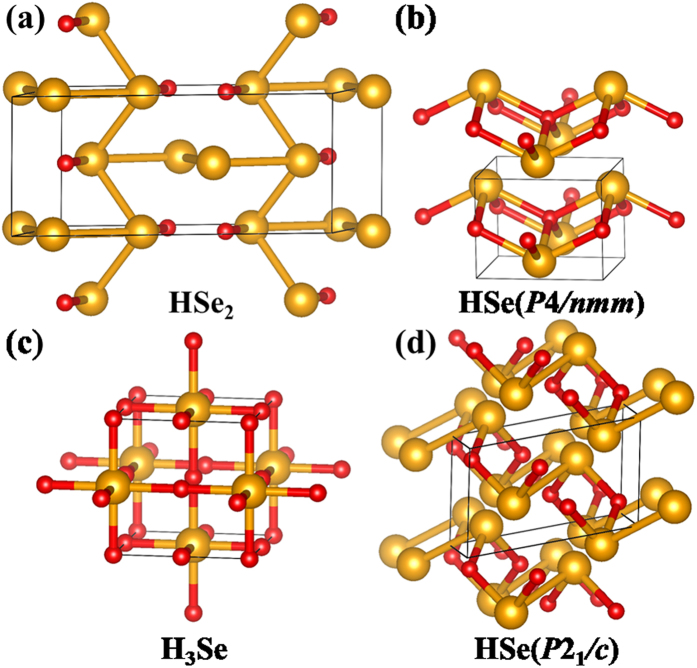
The energetically stable H-Se compounds identified by the structure search: (**a**) HSe_2_ in the *C*2*/m* structure, (**b**) HSe in the *P*4*/nmm* structure and (**c**) H_3_Se in the *Im-*3*m* structure. For the HSe stoichiometry, the metastable *P*2_1_/c structure with competitive enthalpy is shown in (**d**). See Supplementary Table S1 for their detailed structural information and Fig. S2 for more metastable structures and Figs S5–S7 for specific enthalpy-pressure relationship of each stoichiometry.

The Se-rich HSe_2_ stoichiometry is the most stable phase at 200 GPa. Its lowest-energy structure (Fig. 2a) has a *C*2*/m* symmetry, in which the sublattice of Se atoms is isostructural to the Se-IV phase^30^ and H atoms passivate alternatively from both sides of the infinite zigzag Se chains. It is stabilized above 124 GPa (as in the inset of Fig. 1). The HSe stoichiometry is stabilized above 249 GPa as a highly symmetric PbO-type structure (space group *P*4/*nmm*, Fig. 2b). This compound, which is isostructural to superconductive Fe-based chalchogenides^31^, consists of the stack of two-dimensional layered edge-sharing SeH_4_-tetrahedra networks. Within the layer both Se and H are four-fold coordinated. It is worth mentioning that in addition to the *P*4/*nmm* HSe phase, our structure search finds an energetically competitive *P*2_1_/*c* structure (though metastable, Fig. 2d). It consists of layered three-fold coordinated Se/H networks. As to the H_3_Se stoichiometry (stable above 166 GPa), the lowest-energy structure has a high symmetry of *Im-*3 *m*, isostructural to that of H_3_S^17^, where Se atoms occupy a body-centered cubic sublattice, each Se being six-fold coordinated by H. This structure is transformed from a molecular *R*3m phase as the result of pressure-induced hydrogen-bond symmetrization.

We then studied the electronic, phonon and electron-phonon coupling (EPC) properties for the lowest-energy structures of three stable stoichiometries. The band structures of them all exhibit metallic features in their stable pressure regions (see Fig. S3). Phonon calculations indicate their lattice dynamical stabilities evidenced by the absence of any imaginary phonon mode in the whole Brillouin zone.

**Figure 3:**
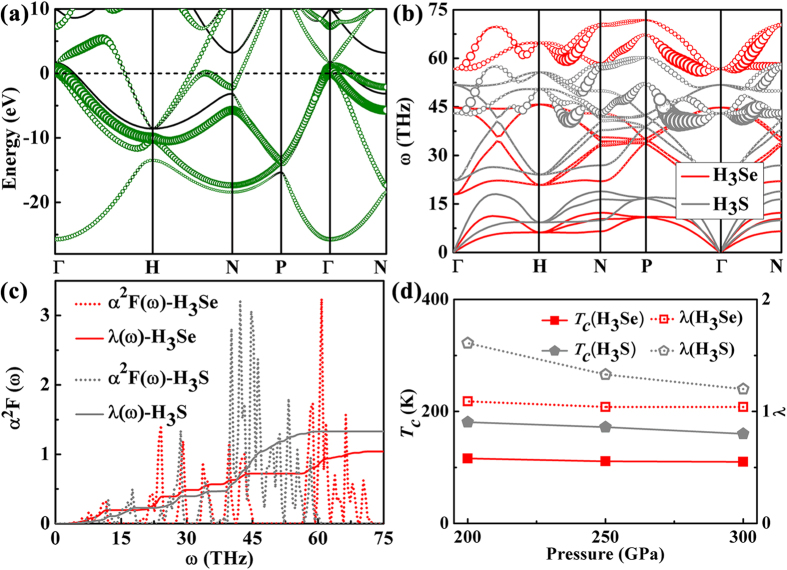
(**a**) Electronic band structure of H_3_Se in the *Im-*3 *m* structure at 250 GPa. The projection onto the H-*s* orbital is depicted by the sizes of green circles. (**b**) Comparison of phonon spectra of H_3_Se (red) and H_3_S (gray) at 250 GPa. The phonon linewidth *γ*_*q,j*_(*ω*) of each mode (*q, j*) caused by EPC is illustrated by the size of circle. (**c**) Eliashberg EPC spectral function *α*^2^*F*(*ω*) and EPC integration *λ*(*ω*) of H_3_Se and H_3_S. (**d**) Pressure dependence of the EPC parameter *λ* (right axis) and *T*_c_ (left axis) for H_3_Se and H_3_S. The typical value of the Coulomb pseudopotential μ^*^ = 0.1 is used for calculating *T*_c_.

For the most H-rich H_3_Se stoichiometry, most of conducting states across the Fermi level (*E*_f_) have significant contribution from the H-*s* orbital, hybridizing strongly with the Se-*p* states, and there exists the combination of flat bands and steep bands^32^ in proximity to the *E*_f_ (Fig. 3a). These features, resembling those of isostructural H_3_S^17^, are potentially favorable for strong EPC. By consideration of the heavier atomic weight of Se than that of S, one expects H_3_Se will have the generally lower phonon frequencies than those of H_3_S at the same pressure. This is indeed the case for the low-frequency Se-derived vibrations (below 15 THz) and mid-lying H-derived wagging and bending modes (between 15 and 45 THz) as shown in Fig. 3b. However, for the high-lying H-stretching vibrations (above 50 THz), one observes an opposite behavior, *i.e.* they show the higher frequencies and separate clearly from the mid-lying regime, unlike the situation of H_3_S in which the H-stretching vibrations mix together with the mid-lying phonons. This may be attributed to the stronger H-Se covalent bond as the result of the larger chemical precompression effect^12^ induced by Se with the larger atomic radius. The calculated phonon linewidths (Fig. 3b) and EPC spectral function (Fig. 3c) indicate a similar mechanism of EPC to that of H_3_S, where the high-frequency H-stretching modes give the notable contribution (31%) to the integral EPC parameter *λ*. This mechanism is different from the cases of superconducting CaH_6_^16^ and SnH_4_^33^ containing quasi-molecular H-units, where the mid-lying H-derived vibrations contribute most significantly to the EPC. For both of compounds, the EPC shows strong anisotropy along different phonon momentum vectors. Because of the hardening of H-stretching phonons, H_3_Se shows a slightly reduced *λ* (1.04 compared with 1.33 of H_3_S), but still falling in the range of fairly strong EPC. Note that owing to the choice of relatively large broadening parameters in our calculations (see Supplementary Methods), our calculated *λ* of H_3_S is a rather conservative evaluation, smaller than the previously reported values, ~2.0^22^,^27^,^34^,^35^. It is thus highly possible that the calculated *λ* of H_3_Se here is underestimated in a similar way. Different from the linearly decreased *λ* with pressure in H_3_S, the *λ* of H_3_Se shows negligible pressure dependence (Fig. 3d). By substituting *λ* into the Allen-Dynes modified McMillan equation^36^, we get a weakly pressure-dependent superconducting *T*_*c*_ around 110 K for H_3_Se, mildly lower than the values of 160–170 K for H_3_S (see also Table S3).

**Figure 4:**
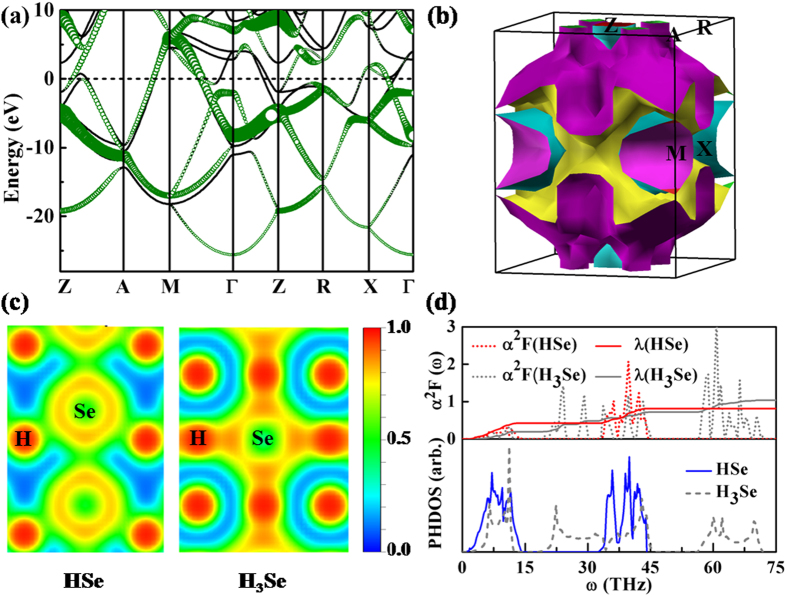
(**a**) Electronic band structure of HSe in the *P*4*/nmm* structure at 250 GPa. Similar to Fig. 3a, the projection onto the H-*s* orbital i*s* indicated by green circles. (**b**) Fermi surface of HSe at 250 GPa. (**c**) Comparison of the electron localization function within the (010) plane of HSe (left) and H_3_Se (right) at the same pressure. (**d**) Phonon density of states (lower panels), Eliashberg EPC spectral function *α*^2^*F*(ω) and EPC integration λ(ω) (upper panels) of HSe and H_3_Se.

Turning to the HSe stoichiometry, there is moderate contribution of the H-*s* state to the conducting states, *i.e.* only several bands across the *E*_f_ (*e.g.* along the A-M, M-Γ and X-Γ lines) are derived from the H-*s* orbital (Fig. 4a). The Fermi surface (Fig. 4b and Fig. S8) consists of the relatively small pockets around the M and Z points, and two expanding sheets at the larger wave-vectors. No notable Fermi nesting can be observed. Analysis of bonding feature via the electron localization function (ELF, Fig. 4c) indicates a much weaker H-Se covalent bond (with the maximum ELF magnitude of ~0.5) by comparison with that of H_3_Se (with the maximum ELF of ~0.9). This is further supported by the elongated bond length of HSe (1.69 Å) than that of H_3_Se (1.51 Å) at the same pressure (300 GPa). The weakening of the covalent H-Se bonding results in a remarkably softening of phonon spectrum (solid blue line in the phonon density of states plot of Fig. 4d) compared with the case of H_3_Se (black dash line). The EPC calculations (the upper panel of Fig. 4d) gives a moderately strong EPC parameter *λ* of ~0.8 (see also Table S3), where the H-derived vibrations make a ~48% contribution. The smaller magnitude of *λ* in HSe than that of H_3_Se can be rationalized by the weakened bonding strength in the covalent-bond system^37^. Meanwhile, due to the relatively low logarithmic averaged phonon frequency *ω*_*log*_ (800–900 K) as the result of phonon softening, HSe exhibits a moderate *T*_*c*_ around 40 K.

For the Se-rich HSe_2_ stoichiometry, as expected its electronic structure is predominated by Se rather than H. The calculated rather small *λ* of 0.45 and low *T*_*c*_ of ~5 K (at 300 GPa) is reminiscent of superconducting solid Se at high pressures^38^.

## Discussion

After the completion of our work, we were aware of the work by Flores-Livas *et al.*^35^ predicting high-temperature superconductivity (with *T*_*c*_ up to 131 K) in selenium hydrides. Our work is different from theirs in several aspects: (i) they only focus on the H_3_Se stoichiometry in analogy to H_3_S, whereas we investigated the entire energy landscape of H-Se system by a more comprehensive global structure search. In addition to H_3_Se, we identified two new energetically stable stoichiometries *i.e.*, HSe_2_ and HSe; (ii) we got a qualitatively different picture of energetic stability for selenium hydrides with respect to the elemental decomposition from theirs. Most of their structures are highly stable relative to the elemental decomposition, but in our work the stable stoichiometries against the elemental decomposition can only emerge at quite high pressures (above 100 GPa); (iii) in contrast to their work assuming H_3_Se as the most stable stoichiometry, we find in fact H_3_Se is marginally stable, and HSe_2_ and HSe are the most stable species at medium and high pressure region, respectively. The implication of our results is that the synthesis of H_3_Se in experiments may require particular kinetic control process.

The HSe phase in the *P*4/*nmm* symmetry represents another interesting high-symmetry structure in addition to the intriguing *Im-*3*m* structure discovered originally in H_3_S^17^. It should be pointed out that this structure is completely different from the one of HS (in the low symmetry of *C*2*/m*) predicted by Errea *et al.*^34^. For such a highly symmetric structure, the transition barrier between it and other isomers is usually high, which points to a strong kinetic stability with respect to variations of external conditions, thus favorable to experimental synthesis.

It should be mentioned that since the screened Coulomb repulsion parameter μ* (in the Allen-Dynes modified McMillan equation) cannot be evaluated by any first principle method, our theoretically predicted *T*_c_ has an intrinsic uncertainty originating from the empirical choice of μ*. Depending on particular materials, μ* usually falls in the range between 0.1 and 0.2^39^. In current study the typical value μ* = 0.1 is used. For completeness, we have calculated the *T*_c_ values of H_3_Se and HSe (at 250 GPa) by taking a series of μ* = 0.05, 0.10, 0.15 and 0.20, as shown in Fig. S10. As seen, as μ* increases from 0.1 to 0.13, a somehow ubiquitous value for metallic hydrides^12^, *T*_*c*_ of H_3_Se and HSe change slightly from ~110 K and ~39 K to ~96 K and ~32 K, respectively.

## Conclusion

In summary, with the aim of finding stable and metallic selenium hydrides for potential high-*T*_*c*_ superconductors, we explore via a global-minimum structure search method the hitherto unknown energy landscape of H-Se system at high pressures. Despite of similar electronic properties of Se and S, the high-pressure phase diagram of H-Se system is distinct from its H-S analogy. The H_2_Se stoichiometry expected from the normal valence states of H (+1) and Se (−2) is surprisingly unstable against the elemental decomposition. Three energetically stable phases, *i.e.* HSe_2_, HSe and H_3_Se, are identified above 120 GPa. While the H_3_S stoichiometry dominates as the most stable phase in the H-S system, H_3_Se turns out to be marginally stable and the most stable stoichiometry is the highly symmetric HSe. All of stable phases exhibit metallic features and superconducting activities. The latter two are predicted to have a high *T*_*c*_ of 40 K (HSe) and 110 K (H_3_Se). Experimental attempt to synthesize these new phases and verification of their superconductivity are called for.

## Methods

The energetic stability of H-Se system is investigated by globally minimizing the potential energy surface at varied stoichiometries via an in-house developed swarm-intelligence based CALYPSO method^40^,^41^ in combination with *ab initio* density functional theory (DFT) total-energy calculations. Its validity in rapidly finding the stable ground-state structures has been demonstrated by its applications in various material systems ranging from elements to binary and ternary compounds^40^,^42^,^43^,^44^. The energetic calculations are performed using the plane-wave pseudopotential method within the generalized gradient approximation through the Perdew–Burke–Ernzerhof (PBE) exchange-correlation functional^45^, as implemented in the VASP code^46^. The electron-ion interaction was described by the projected-augmented-wave potentials with 1*s*^1^ and 4*s*^2^4*p*^4^ as valence electrons for H and Se, respectively. During the structure search, an economy set of parameters are used to calculate the relative energetics of sampled structures, following which the cutoff energy of 600 eV for the expansion of wave-function and Monkhorst−Pack *k*-point sampling with grid spacing of 2π × 0.03 Å^−1^ were chosen to ensure the enthalpy converged to better than 1 meV/atom. The validity of pseudopotentials used at high pressures is carefully examined with the full-potential linearized augmented plane-wave method through the WIEN2k package^47^. The phonon spectrum for evaluating the lattice dynamic stability and electron-phonon coupling for superconducting properties of stable phases are performed within the framework of the linear-response theory via Quantum-ESPRESSO package^48^. The spin-orbit coupling is found to have a negligible effect on the electronic and superconductive properties of the current system (Fig. S11 and Table S4), and is thus reasonably neglected. See Supplementary information for more details.

## Additional Information

**How to cite this article**: Zhang, S. *et al.* Phase Diagram and High-Temperature Superconductivity of Compressed Selenium Hydrides. *Sci. Rep.* **5**, 15433; doi: 10.1038/srep15433 (2015).

## Supplementary Material

Supplementary Information
