# Supplementary material for: Phase Diagram and High-Temperature Superconductivity of Compressed Selenium Hydrides
Source: Sci Rep. 2015 Oct 22;5:15433. doi: 10.1038/srep15433 (PMC4614537; doi:10.1038/srep15433)
Supplement: Supplementary Information [file srep15433-s1.pdf]

Supplementary Information for the paper entitled "*Phase Diagram and High-Temperature Superconductivity of Compressed Selenium Hydrides*"

Shoutao Zhang[1], Yanchao Wang[1], Jurong Zhang[1], Hanyu Liu[1], Xin Zhong[1], Hai-Feng Song[4,5], Guochun Yang[2,1], Lijun Zhang[3,1], Yanming Ma[1]

[1]*State Key Laboratory of Superhard Materials, Jilin University, Changchun 130012, China.*
[2]*Faculty of Chemistry, Northeast Normal University, Changchun 130024, China.*
[3]*College of Materials Science and Engineering and Key Laboratory of Automobile Materials of MOE, Jilin University, Changchun 130012, China*
[4]*LCP, Institute of Applied Physics and Computational Mathematics, Beijing 100088, China.*
[5]*Software Center for High Performance Numerical Simulation, China Academy of Engineering Physics, Beijing 100088, China.*

Supplementary Methods

Our structural prediction approach is based on a global minimization of free energy surfaces of given compounds by combining *ab initio* total-energy calculations with the particle swarm optimization (PSO) algorithm.[1,2] The structure search of each H_xSe_y (x = 1−5 and y = 1−3) stoichiometry is performed with simulation cells containing 1−4 formula units. In the first generation, a population of structures belonging to certain space group symmetries are randomly constructed. Local optimizations of candidate structures are done by using the conjugate gradients method through the VASP code[3], with an economy set of input parameters and an enthalpy convergence threshold of 1×10^{-5} eV per cell. Starting from the second generation, 60% structures in the previous generation with the lower enthalpies are selected to produce the structures of next generation by the PSO operators. The 40% structures in the new generation are randomly generated. A structure fingerprinting technique of bond characterization matrix is employed to evaluate each newly generated structure, so that identical structures are strictly forbidden. These procedures significantly enhance the diversity of sampled structures during the evolution, which is crucial in driving the search into the global minimum. For most of cases, the structure search for each chemical composition is converged (evidenced by no structure with the lower enthalpy emerging) after 1000 ~ 1200 structures investigated (*i.e.* in about 20 ~ 30 generations).

The energetic stabilities of different H_xSe_y stoichiometries are evaluated by their formation enthalpies relative to the products of dissociation into constituent elements (*i.e.* solidified phases of H_2 and Se):

$$\Delta H = [h(H_xSe_y) - xh(H) - yh(Se)]/(x+y) \quad (1)$$

where h represents absolute formation enthalpy. By regarding H and Se as the binary variables, with these ΔH values we can construct the convex hull at each pressure (Fig. 1in the main text). It is known that the zero-point energy plays an important role in determining the phase stabilities of the compounds containing light elements such as

H. We hence examine the effect of zero-point energy on the stability of the stoichiometries on the convex hull (Fig. S9), by using the calculated phonon spectrum with the supercell approach[6] as implemented in the Phonopy code[7].

The electron-phonon coupling calculations are carried out with the density functional perturbation (linear response) theory as implemented in the QUANTUM ESPRESSO package.[8] We employ the norm-conserving pseudopotentials with $1s^1$ and $4s^2 4p^4$ as valence electrons for H and Se. The kinetic energy cutoff for wave-function expansion is chosen as 70 Ry. To reliably calculate electron-phonon coupling in metallic systems, we need to sample dense *k*-meshes for electronic Brillouin zone integration and enough *q*-meshes for evaluating average contributions from the phonon modes. Dependent on specific structures of stable compounds, different *k*-meshes and *q*-meshes are used: 16 x 16 x 16 *k*-meshes and 4 x 4 x 4 *q*-meshes for HSe_2 in the *C*2/*m* structure, 18 x 18 x 24 *k*-meshes and 3 x 3 x 6 *q*-meshes for HSe with the *P*4/*nmm* structure, 24 x 24 x 24 *k*-meshes and 6 x 6 x 6 *q*-meshes H_3Se with the *Im*-3*m* structure. Note that the calculated EPC strength is affected by two critical parameters associated with the broadening technique: one broadening parameter (δ) is to account for the Fermi surface of metals; the other broadening parameter (σ) is for evaluating the integration of the double-delta function within the expression of phonon linewidth. In principle, the values of δ and σ can be chosen as small as possible (approaching to zero) when the dense enough *k*-meshes are used. This ideal case is difficult to be achieved since the finite *k*-meshes are always adopted in the calculations. Here for safety we used the relatively large values of δ (0.05 Ry) and σ (0.04 Ry). They thus give a rather conservative evaluation of total EPC constant λ.

To examine the reliability of the adopted projected-augmented-wave (PAW) pseudopotentials for H and Se, the formation enthalpy calculations of H_3Se with other types of pseudopotentials (contained in the VASP code) are performed. The results are shown in Table S0. As seen different pseudopotentials generally give consistent results, though the LDA ones turns out to overcount the magnitude of formation enthalpy. The reliabilities of the pseudopotentials at high pressures are also crosschecked with the full-potential linearized augmented plane-wave (LAPW)

method as implemented in the WIEN2k code[9]. By using the two different methods, we calculate total energies of H_3Se in the *Im*-3*m* structure with varying pressures, and then fit the obtained energy-volume data into the Birch-Murnaghan equation of states. Figure S0 shows the resulted fitted equation of states. We can see the results derived from two methods are almost identical, which clearly indicates the suitability of the PAW pseudopotentials for describing the energetics of Se hydrides at megabar pressures.

Table S0. Calculated formation enthalpy (in eV) per formula unit of H_3Se with respect to solid H_2 and Se using different PAW pseudopotentials at 150 GPa.

**PBE**	**H**	**H_s**	**H_h**	**H_GW**
**Se**	-0.0092	-0.0193	-0.0105	-0.0105
**Se_GW**	-0.0079	-0.0180	-0.0093	-0.0093
**LDA**	**H**	**H_s**	**H_h**	**H_GW**
**Se**	-0.1531	-0.1538	-0.1536	-0.1538
**Se_GW**	-0.1528	-0.1534	-0.1533	-0.1534

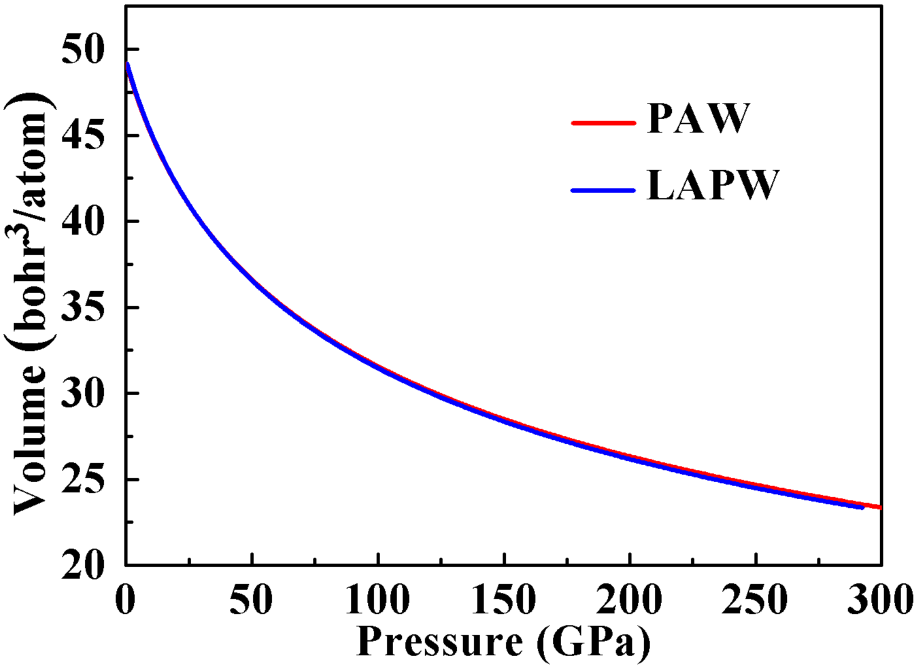

Figure S0. Comparison of the fitted Birch-Murnaghan equation of states for H_3Se in the *Im*-3*m* structure by using the calculated results from the PAW pseudopotentials and the full-potential LAPW methods.

Supplementary Figures

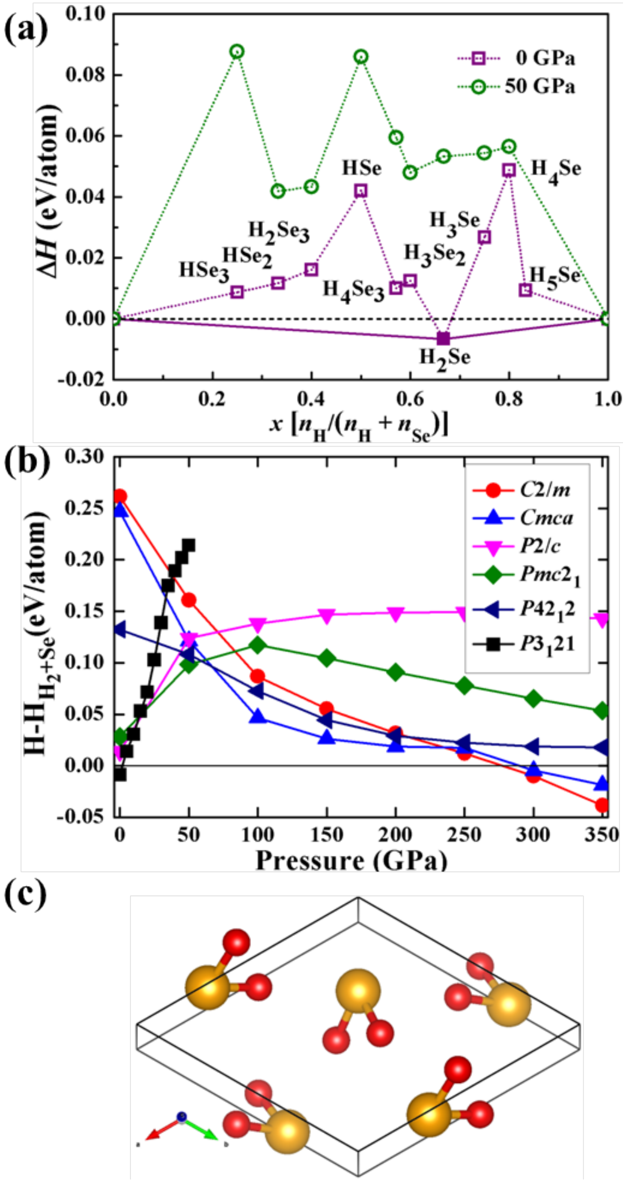

Figure S1. (a) Phase stabilities of various H-Se compounds at the low pressure range of 0 and 50 GPa. The formation enthalpies of H-Se compounds are relative to the enthalpies of elemental decomposition into solidified phases of H_2 and Se. Dashed lines connect data points, and solid lines denote the convex hull. Compounds corresponding to data points located on the convex hull are stable against decomposition. (b) Calculated enthalpies per atom of various structures for stoichiometry H_2Se in the pressure range of 0–350 GPa with respect to H_2+Se. (c) Predicted structure of H_2Se with $P3_121$ symmetry at 0 GPa.

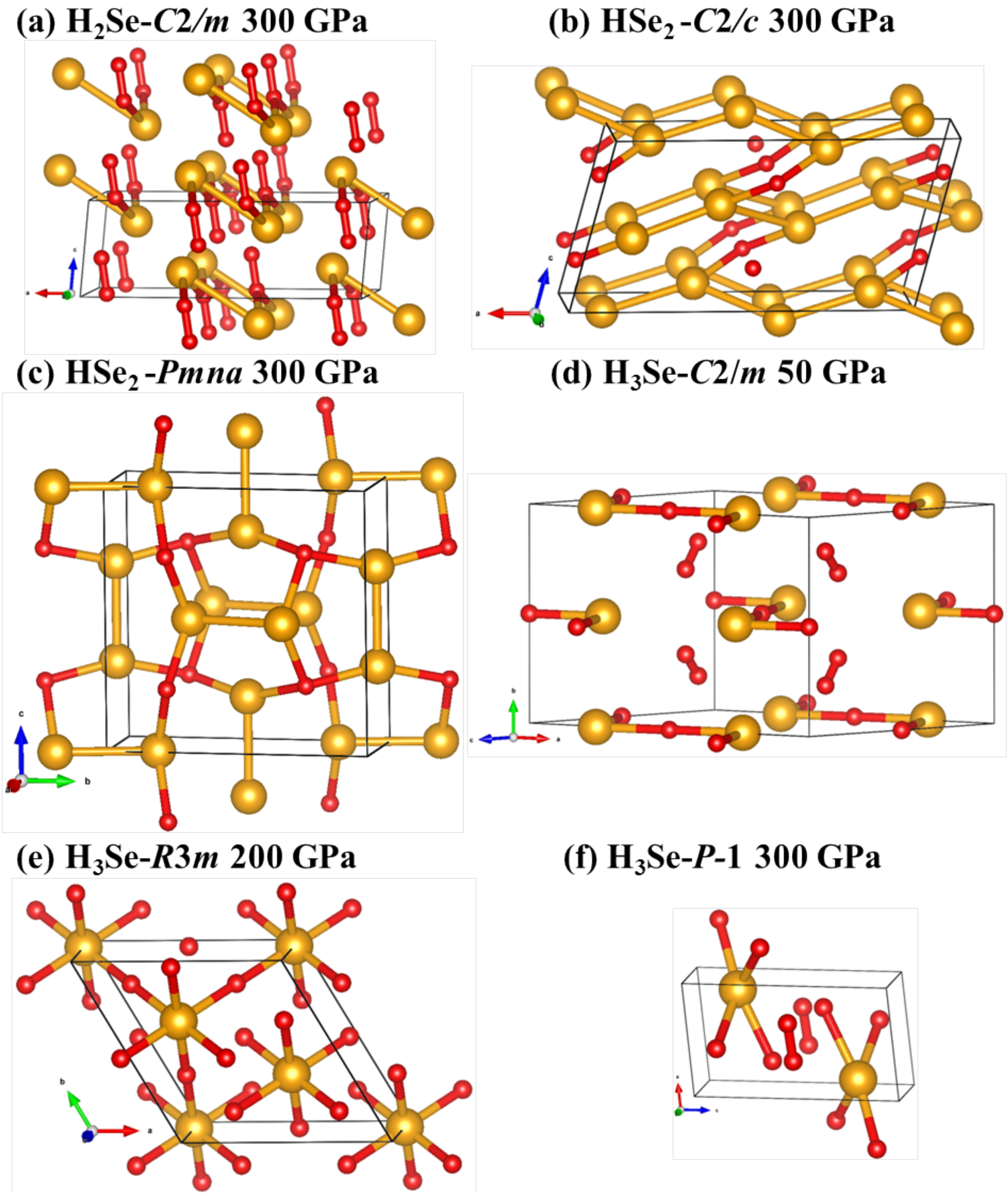

Figure S2. The metastable structures for stoichiometries H_2Se, HSe_2 and H_3Se at selected pressures. The corresponding detailed structural information is summarized in Table S2.

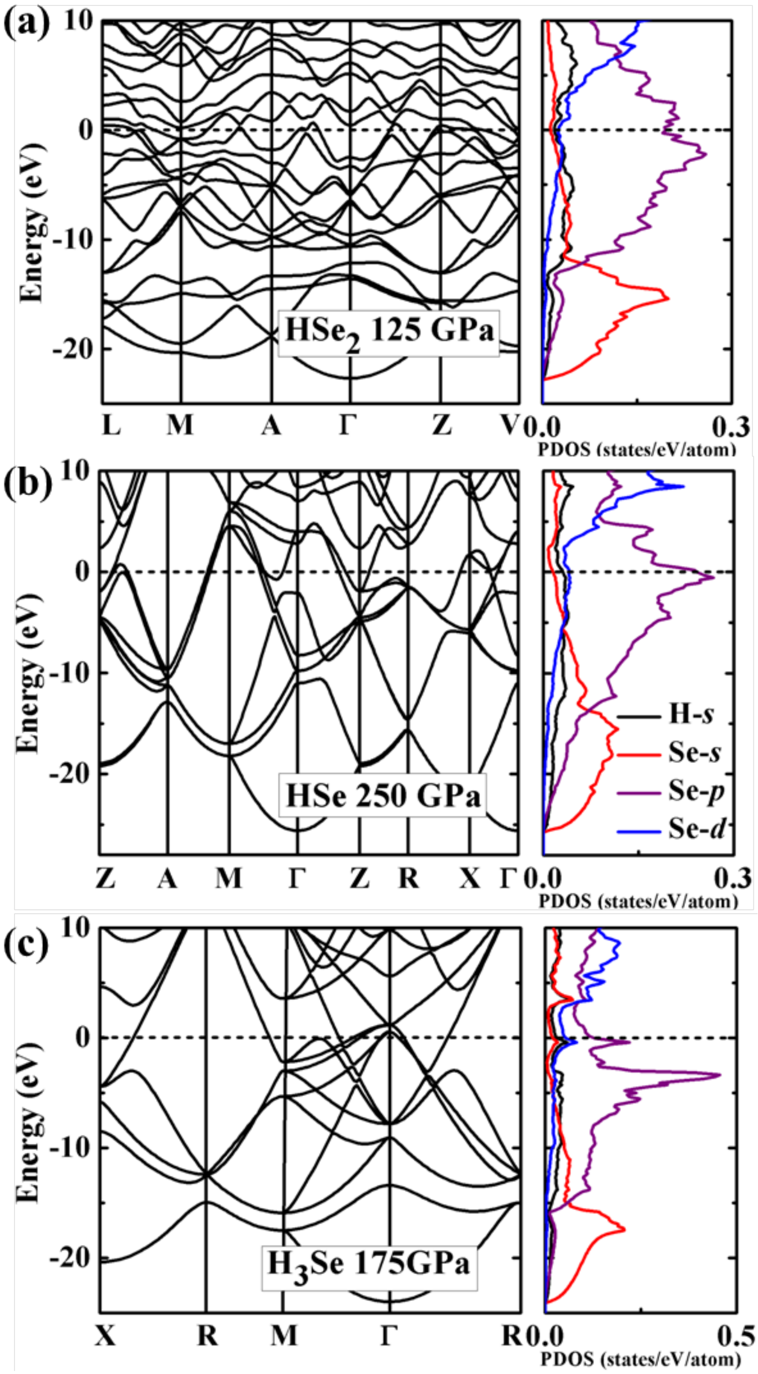

Figure S3. Calculated electronic band structure (left panels) and projected density of states (right panels) for the predicted H-rich compounds at the lower boundary of respective stable pressure region: (a) HSe_2(*C*2/*m*) at 125 GPa, (b) HSe(*P*4/*nmm*) at 250 GPa, and (c) H_3Se(*Im*-3*m*) at 175 GPa.

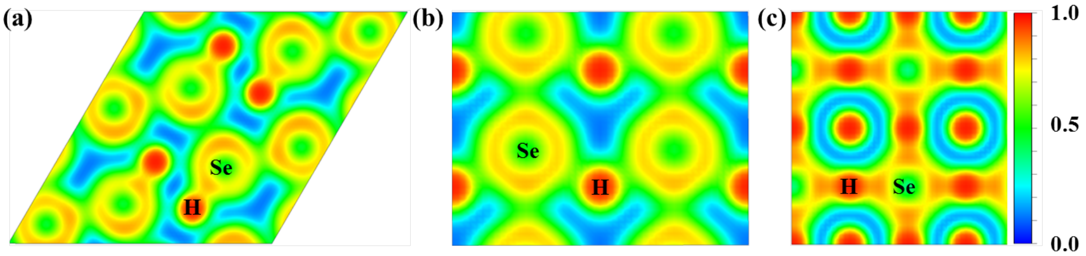

Figure S4. Two-dimensional plots of the electron localization function (ELF) for HSe_2($C2/m$), HSe($P4/nmm$) and H_3Se(Im-3m) at 300 GPa.

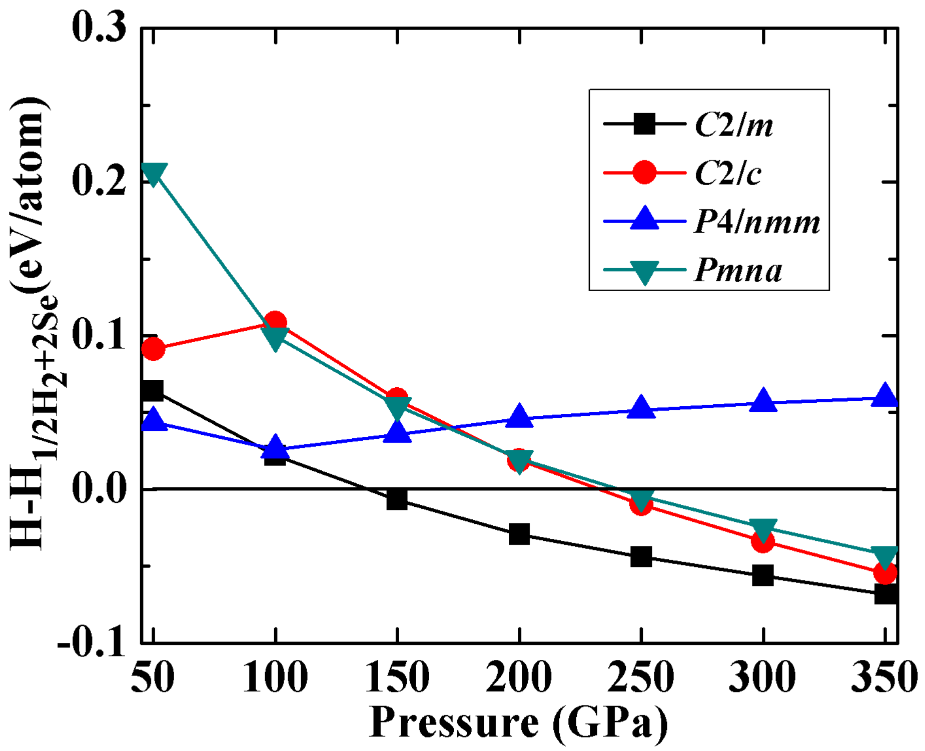

Figure S5. Calculated enthalpies per atom of various structures for stoichiometry HSe_2 in the pressure range of 50–350 GPa with respect to $1/2H_2$+2Se.

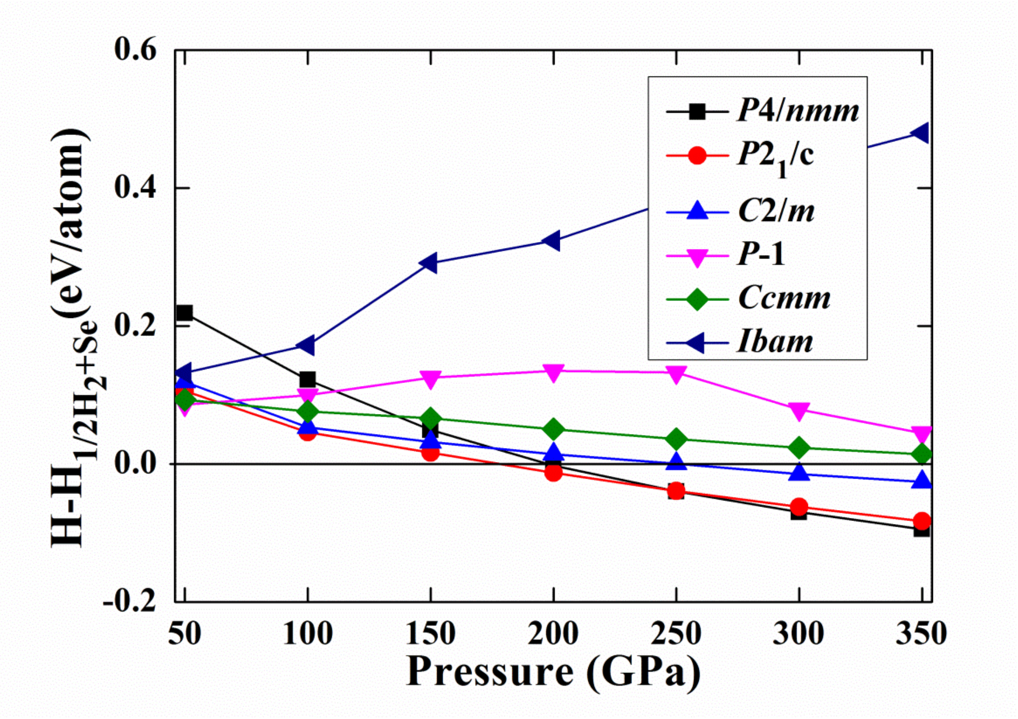

Figure S6. Calculated enthalpies per atom of various structures for stoichiometry HSe as functions of pressure between 50 and 350 GPa with respect to $1/2H_2$+Se.

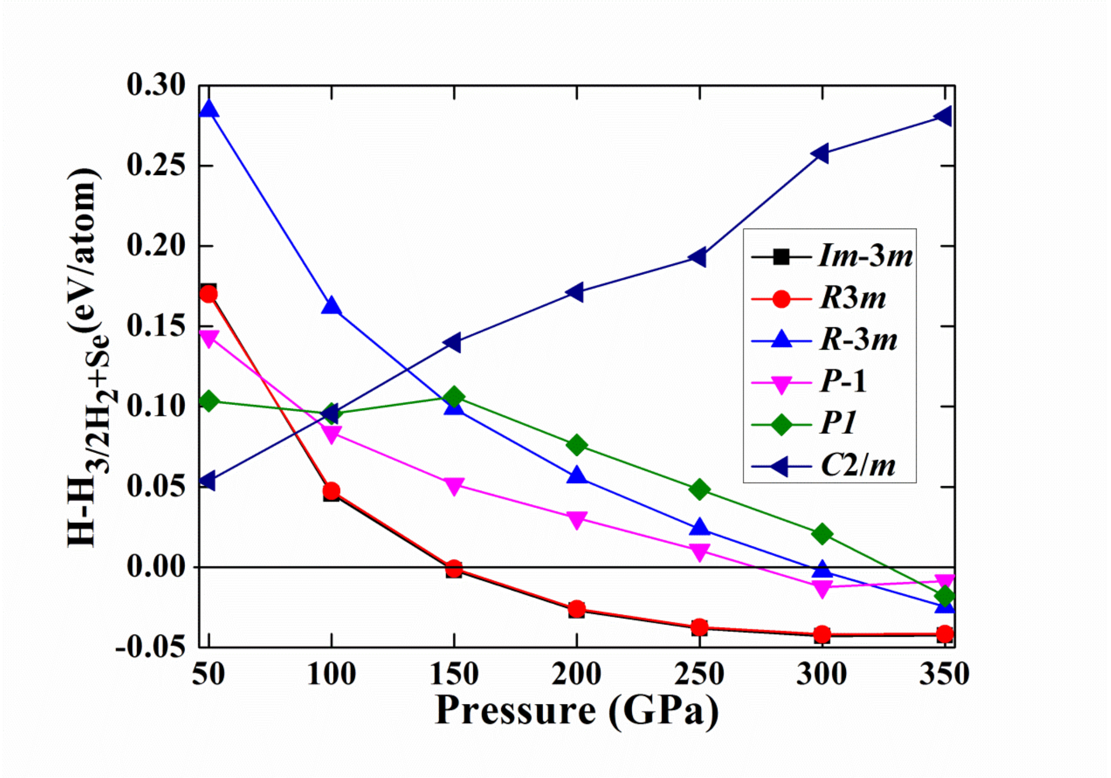

Figure S7. Calculated enthalpies per atom of various structures for stoichiometry H_3Se as functions of pressure between 50 and 350 GPa with respect to $3/2H_2$+Se.

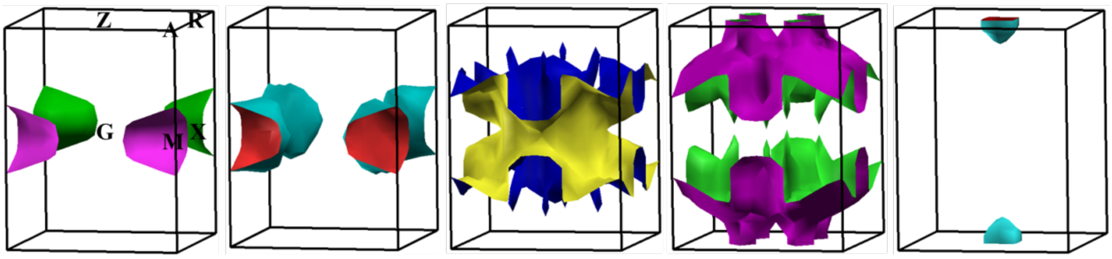

Figure S8. Fermi surfaces for HSe in the *P*4/*nmm* structure at 250 GPa.

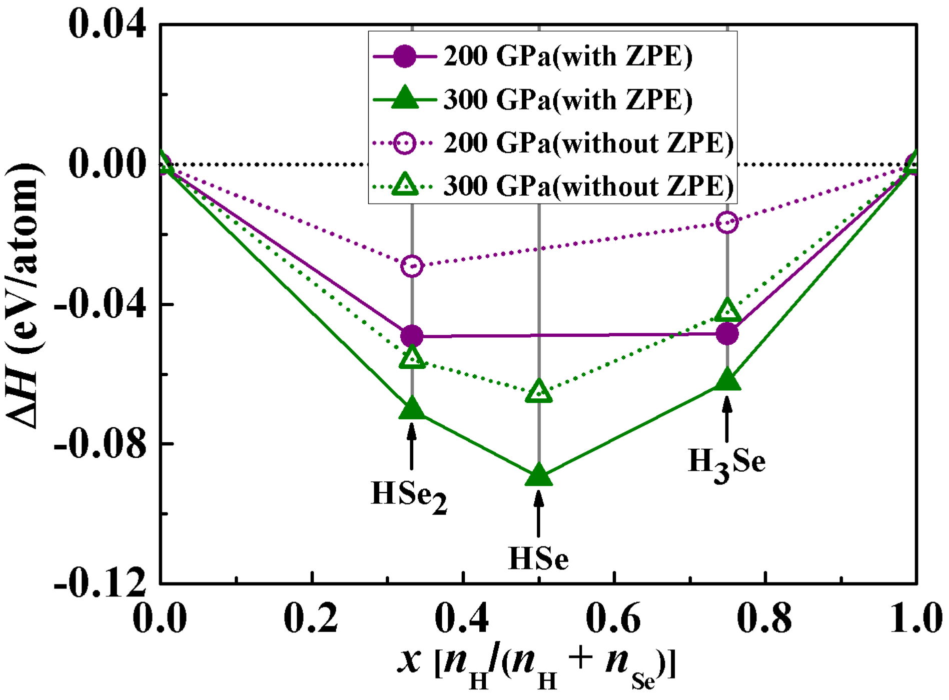

Figure S9. Relative formation enthalpies of the predicted stable stoichiometries, *e.g.* HSe_2, HSe and H_3Se at 200 and 300 GPa with (filled symbols and solid lines) and without (open symbols and dash lines) the inclusion of zero-point energy contribution. One clearly sees that the introduction of zero-point energy does not change energetic stabilities of these stable stoichiometries.

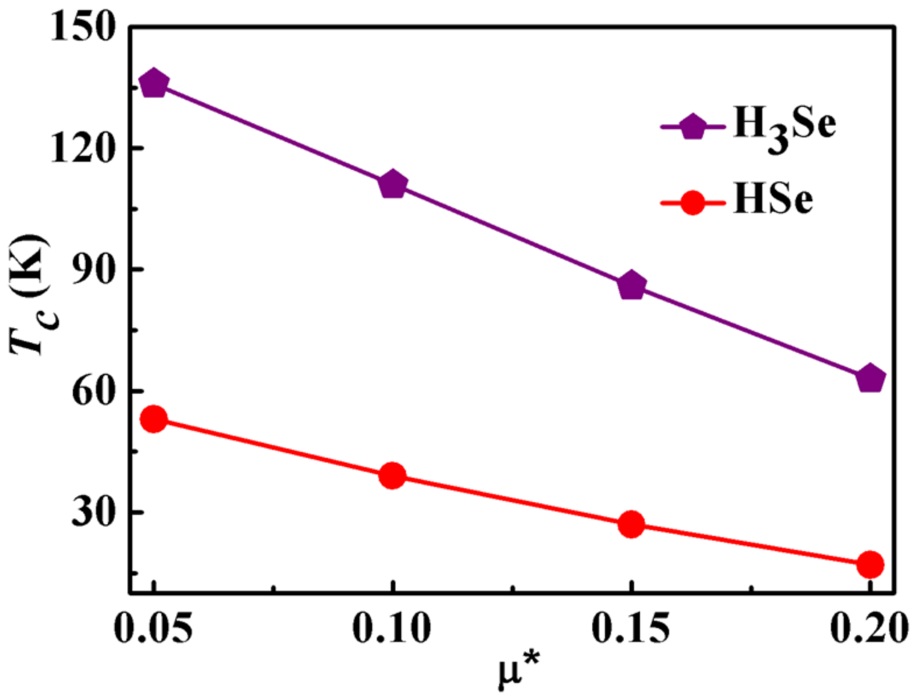

Figure S10. The dependence of T_c on μ^* for H_3Se(*Im*-3*m*) and HSe(*P*4/*nmm*) at 250 GPa.

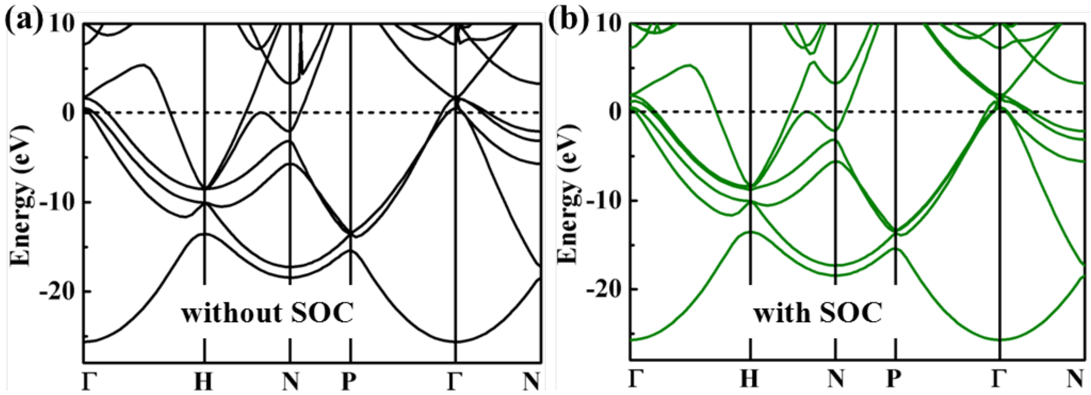

Figure S11. Calculated electronic band structure for H_3Se(*Im*-3*m*) at 250 GPa without (a) and with (b) the inclusion of spin-orbit coupling (SOC) contribution.

Supplementary Tables

Table S1. Detailed structural information of the predicted stable H-Se compounds at selected pressures.

Phases	Pressure (GPa)	Lattice parameters (Å, °)	Atomic coordinates (fractional)			
**HSe_2-*C2/m***	300	$a = 7.580$	H(4i)	0.376	0.000	0.288
		$b = 3.222$	Se(4i)	0.105	0.000	0.842
		c = 3.858	Se(4i)	0.355	0.000	0.672
		$a = \gamma = 90.000$				
		$\beta = 120.320$				
**HSe-*P4/nmm***	300	$a = b = 3.014$	H(2b)	0.500	0.500	0.500
		c = 2.373	Se(2c)	0.500	0.000	0.817
		$\alpha = \beta = \gamma = 90.000$				
**H_3Se-*Im*-3*m***	300	$a = b = c = 3.025$	H(6b)	0.500	0.000	0.500
		$\alpha = \beta = \gamma = 90.000$	Se(2a)	0.500	0.500	0.500
**HSe_2-*C2/m***	200	$a = 7.897$	H(4i)	0.376	0.000	0.288
		$b = 3.310$	Se(4i)	0.105	0.000	0.842
		$c = 4.048$	Se(4i)	0.355	0.000	0.672
		$\alpha = \gamma = 90.000$				
		$\beta = 120.341$				
**H_3Se-*Im*-3*m***	200	$a = b = c = 3.148$	H(6b)	0.500	0.000	0.500
		$\alpha = \beta = \gamma = 90.000$	Se(2a)	0.500	0.500	0.500
**H_2Se-$P3_121$**	0	$a = b = 5.873$	H(6c)	0.900	0.450	0.030
		$c = 6.871$	Se(3b)	1.320	0.320	0.500
		$\alpha = \beta = 90.000$				
		$\gamma = 120.000$				

Table S2. Detailed structural information of the predicted metastable H-Se compounds at selected pressures.

Phases	Pressure (GPa)	Lattice parameters (Å, °)	Atomic coordinates (fractional)			
**H_2Se-*C2/m***	300	a = 6.983	H(4i)	0.064	0.000	0.596
		b = 2.937	H(4i)	0.094	0.000	0.965
		c = 2.389	Se(4i)	0.144	0.500	0.261
		$\alpha = \gamma$ = 90.000				
		β = 95.442				
**HSe_2 -*C2/c***	300	a = 8.253	H(4e)	0.000	0.523	0.250
		b = 2.363	Se(8f)	0.132	0.184	0.089
		c = 4.286				
		$\alpha = \gamma$ = 90.000				
		β = 104.475				
**HSe_2 -*Pmna***	300	a = 5.899	H(4g)	0.250	0.200	0.250
		b = 2.342	Se(4f)	0.312	0.500	0.000
		c = 5.869	Se(4h)	0.000	0.890	0.187
		$\alpha = \beta = \gamma$ = 90.000				
**H_3Se-*C*2/*m***	50	a = 6.476	H(2b)	0.000	0.500	0.000
		b = 4.875	H(2c)	0.500	0.500	0.500
		c = 4.503	H(8j)	0.220	0.194	0.420
		$\alpha = \gamma$ = 90.000	Se(4i)	0.259	0.000	0.033
		β = 136.386				
**H_3Se-*R*3*m***	200	$a = b$ = 4.451	H(9b)	0.500	0.500	0.298
		c = 2.728	Se(3a)	0.000	0.000	0.298
		$\alpha = \beta$ = 90.000				
		γ = 120.000				
**H_3Se-*P*-1**	300	a = 2.588	H(2i)	0.335	0.010	0.505
		b = 2.565	H(2i)	0.277	0.623	0.371
		c = 4.279	H(2i)	0.422	0.948	0.130
		α = 91.920	Se(2i)	0.865	0.349	0.217
		β = 94.937				
		γ = 73.627				
**HSe-*P*2_1*/c***	300	a = 4.081	H(1a)	0.924	0.412	0.193
		b = 4.456	H(1a)	0.075	0.588	0.806
		c = 2.563	H(1a)	0.575	0.912	0.306
		$\alpha = \gamma$ = 90.000	H(1a)	0.424	0.088	0.693
		β = 78.453	Se(1a)	0.697	0.640	0.631
			Se(1a)	0.302	0.359	0.368
			Se(1a)	0.802	0.140	0.868
			Se(1a)	0.197	0.859	0.131

Table S3. Calculated electron-phonon coupling parameter λ, logarithmic average frequency $\omega_{\log}$, electronic density of states at the Fermi level N (Ef) and critical temperature T_c of predicted H-Se compounds and H_3S.

**Phases**	**Pressure (GPa)**	**λ**	**$\omega_{\log}$ (K)**	**$N(E_f)$ states/spin/Ry/cell**	**T_c (K)**
**HSe_2-*C2/m***	300	0.45	647	8.09	5
**HSe-*P*2$_1$*/c***	300	0.65	832	9.55	23
**HSe-*P4/nmm***	250	0.81	813	5.62	39
	300	0.80	885	5.75	42
**H_3Se-*Im*-3*m***	200	1.09	1477	3.19	116
	250	1.04	1498	3.13	111
	300	1.04	1492	3.09	110
**H_3S-*Im*-3*m***	200	1.61	1484	3.27	171
	250	1.33	1708	3.25	172
	300	1.20	1774	3.23	160

Table S4. Calculated electron-phonon coupling parameter λ, logarithmic average frequency $\omega_{\log}$, electronic density of states at the Fermi level N (Ef) and critical temperature T_c for H_3Se(*Im*-3*m*) at 250 GPa with/without the inclusion of spin-orbit coupling (SOC) contribution.

**Phases**		**λ**	**$\omega_{\log}$ (K)**	**$N(E_f)$ states/spin/Ry/cell**	**T_c (K)**
**H_3Se-*Im*-3*m***	**non-SOC**	1.04	1498	3.13	111
	**SOC**	1.10	1386	3.13	111

References

[1] Wang, Y., Lv, J., Zhu, L. & Ma, Y. Crystal structure prediction via particle-swarm optimization. *Phys. Rev. B,* **82**, 094116 (2010).

[2] Wang, Y., Lv, J., Zhu, L. & Ma, Y. CALYPSO: A method for crystal structure prediction. *Comput. Phys. Commun.* **183**, 2063-2070 (2012).

[3] Kresse, G. & Furthmuller, J. Efficient iterative schemes for ab initio total-energy calculations using a plane-wave basis set. *Phys. Rev. B,* **54**, 11169-11186 (1996).

[4] Perdew, J.P. et al. Atoms, molecules, solids, and surfaces: applications of the generalized gradient approximation for exchange and correlation. *Phys. Rev. B,* **46**, 6671-6687 (1992).

[5] Feng, J., Hennig, R. G., Ashcroft, N. W. & Hoffmann, R. Emergent reduction of electronic state dimensionality in dense ordered Li–Be alloys. *Nature,* **451**, 445-448 (2008).

[6] Dove, M. T. Introduction to lattice dynamics, cambridge university press. ISBN 0521392934 (1993).

[7] Togo, A., Oba, F. & Tanaka, I. First-principles calculations of the ferroelastic transition between rutile-type and $CaCl_2$-type SiO_2 at high pressures. *Phys. Rev. B* **78**, 134106 (2008).

[8] Giannozzi, P. et al. QUANTUM ESPRESSO: a modular and open-source software Project for quantum simulations of materials. *J. Phys.: Condens. Matter.* **21**, 395502 (2009).

[9]Blaha, P., Schwarz, K., Madsen, G., Kvasnicka, D. & Luitz, J. WIEN2k, An augme nted plane wave + local orbitals program for calculating crystal properties (Karlheinz Schwarz, Techn. Universität Wien, Austria) ISBN 3-9501031-9501031-9501032 (200 1).
